# A 2D-QSAR and Grid-Independent Molecular Descriptor (GRIND) Analysis of Quinoline-Type Inhibitors of Akt2: Exploration of the Binding Mode in the Pleckstrin Homology (PH) Domain

**DOI:** 10.1371/journal.pone.0168806

**Published:** 2016-12-30

**Authors:** Noreen Akhtar, Ishrat Jabeen

**Affiliations:** Research Centre for Modeling and Simulation (RCMS), National University of Sciences and Technology (NUST), Islamabad, Pakistan; Istituto di Genetica Molecolare, ITALY

## Abstract

Protein kinase B-β (PKBβ/Akt2) is a serine/threonine-specific protein kinase that has emerged as one of the most important regulators of cell growth, differentiation, and division. Upregulation of Akt2 in various human carcinomas, including ovarian, breast, and pancreatic, is a well-known tumorigenesis phenomenon. Early on, the concept of the simultaneous administration of anticancer drugs with inhibitors of Akt2 was advocated to overcome cell proliferation in the chemotherapeutic treatment of cancer. However, clinical studies have not lived up to the high expectations, and several phase II and phase III clinical studies have been terminated prematurely because of severe side effects related to the non-selective isomeric inhibition of Akt2. The notion that the sequence identity of pleckstrin homology (PH) domains within Akt-isoforms is less than 30% might indicate the possibility of the development of selective antagonists against the Akt2 PH domain. Therefore, in this study, various *in silico* tools were utilized to explore the hypothesis that quinoline-type inhibitors bind in the Akt2 PH domain. A Grid-Independent Molecular Descriptor (GRIND) analysis indicated that two hydrogen bond acceptors, two hydrogen bond donors and one hydrophobic feature at a certain distance from each other were important for the selective inhibition of Akt2. Our docking results delineated the importance of Lys30 as an anchor point for mapping the distances of important amino acid residues in the binding pocket, including Lys14, Glu17, Arg25, Asn53, Asn54 and Arg86. The binding regions identified complement the GRIND-based pharmacophoric features.

## Introduction

Akt (Protein Kinase B) is a serine/threonine kinase with three structurally homologous mammalian isoforms (Akt1, Akt2 and Akt3) that are respectively encoded by the genes *PKBα*, *PKBβ* and *PKBγ* [1–4]. The activation of Akt facilitates growth factor-mediated cell survival by inhibiting apoptosis via the phosphorylation and inactivation of various pro-apoptotic signals including Bcl-2-associated death (BAD) [5] and Forkhead box O (FOXOs) [6, 7], and promotes cell proliferation by phosphorylation and inhibition of the tumour suppressor tuberous sclerosis complex 2 (TSC2) and the activation of mammalian target of rapamycin complex 1 (mTORC1) [8, 9]. Additionally, it has been reported that Akt is also involved in the activation of various oncogenic signalling pathways such as Nuclear factor kappa B (NF-κB), c-myelocytomatosis (c-Myc), Vascular endothelial growth factor (VEGF) and Cyclin D, thus acting as a central regulator of various cellular functions including cell growth, survival, and metabolism [10].

All three Akt isoforms share a similar structural topology. Each contains a remarkably conserved amino-terminal pleckstrin homology (PH) domain, a central serine/threonine catalytic kinase domain (ATP-binding domain) and a small carboxy-terminal regulatory domain [4, 11]. However, the Akt isoforms differ in their physiological function, tissue distribution and expression in various tumours [12]. For instance, Akt1 and Akt2 are ubiquitously expressed in the liver, pancreas, colon, adipose tissue and skeletal muscle and; are involved in cell growth or survival and glucose homeostasis [13–16]. Akt3 has limited distribution and expression in the central nervous system, the heart, kidneys, lungs and skeletal muscle [16, 17]. Therefore, use of selective inhibitors of these isoforms during cancer therapy is a promising concept to overcome cell proliferation in various tumours. The highly conserved ATP-binding domain of the AGC kinase family is associated with the promiscuous inhibition of the Akt-isoforms and might offer various off-target toxicities [18–20] and thus has proved to be a major hurdle in developing small molecule inhibitors against Akt. To overcome this drawback, targeting the PH domain of Akt to interfere with its binding to phosphatidylinositol 3,4,5 trisphosphate (PIP3) and membrane translocation has been proposed by several researchers in the past [21–24]. Because the sequence identity of the PH domains of the Akt-isoforms is less than 30%, it might be possible to develop selective antagonists against the PH domain of the Akt-isoforms [18, 25].

Several reports have described the overexpression of Akt2 in various human cancers including, prostate, ovarian, breast, and pancreatic [26–28], and thus its role in tumorigenesis [29], poor prognosis [30], and chemo- and radio-therapeutic resistance [31] in cancer patients have been reported in recent investigations.

Akt2 inhibitors have been synthesized and biologically evaluated for their efficacy against the ATP binding domain. Such inhibitors include a series of diphenyl quinoxalines [22], imidazopyridine [32], 2-pyrimidyl-5-amidothiophenes [33], prenylated flavonoids [34, 35], pyrimidines [36] and pyridines [37, 38]. Furthermore, ligand as well as structure-based modelling strategies, including the support vector classification (SVC) method [39], comparative molecular similarity indices analysis (CoMSIA), comparative molecular field analysis (CoMFA) [40–42] and molecular docking simulations [40, 43] have been used for the structural optimization of the identified lead compounds. However, the results of most investigations were disappointing; only a handful of compounds have reached the clinical investigation stage, and none could be marketed for routine clinical usage to circumvent cell proliferation during cancer chemotherapy because of their off-target toxicity. Thus, the development of selective inhibitors of Akt2 by targeting its PH domain may be worthwhile to obtain safer and more efficacious drugs to inhibit cell proliferation in cancer chemotherapy. However, the experimental protocols for the assessment of potency and isoform selectivity are very expensive and, thus, are not well suited for extensive screening. Therefore, reliable *in silico* methods to characterize the interactions of the Akt2 PH domain with small molecular inhibitors would be greatly beneficial and help to reduce late stage failures in clinical studies. The purpose of this study was the identification of specific 3D structural features important for the interaction with the Akt2 PH domain. Grid-Independent Molecular Descriptor (GRIND) models were developed for this purpose on a previously published dataset of quinoline-type inhibitors of Akt2, which are active against the PH domain. In addition, molecular docking of selected compounds in a homology model of Akt2 was done to shed light on the potential binding conformations of these compounds and to develop a specific binding hypothesis for the Akt2 PH domain.

## Methodology

### Dataset

A series of inhibitors active against the Akt2 PH domain was extracted from the literature [44–52]. The dataset is shown in S1 Table, and comprises 111 compounds which consist of pyrido [2, 3-d]-pyrimidine, quinoxaline and pyrido [2, 3-d]-pyrizine-type quinoline derivatives. For the present QSAR study, the reported inhibitory potencies (IC_50_) of the inhibitors range from 0.019 μM to 230 μM. A diverse subset selection method [53, 54] was used to divide the compounds into a training (80%) and test set (20%). Briefly, about 300 2D and 3D descriptors available in the MOE version 2013.0802 software [55] were employed for the distance calculation of each database entry. The test set comprised 20% of the data structures (22 compounds) that had greater distance values from each other and the remaining 89 compounds (80%) were taken as the training set.

### Calculation of Physicochemical Parameters (2D-QSAR)

The molecular builder function in MOE version 2013.0802 was used to build 3D structures of the compounds in the dataset [55], and their energy was minimized by the MMFF94 force field [56]. For the Hansch analysis, 2D physicochemical descriptors such as lipophilicity, polarizability, electronic and steric parameters were calculated with MOE version 2013.0802 [55]. Furthermore, a QuaSAR contingency descriptor selection program [57] was used to extract the most relevant molecular descriptors for the training set. A partial least squares analysis using a leave-one-out (LOO) method [58] was used to correlate the most important molecular descriptors with the inhibitory potency log(1/IC_50_) values. To verify robustness, an external test set of 20% compounds was used to validate the final model.

### Homology Modelling

Because no crystal structure for the the Akt2 PH domain was available, a homology modelling technique was adopted to model its 3D structure. The amino acid sequence of the Akt2 PH domain (P31751) was retrieved from the UniProt database [59]. The crystal structure of PH domain of Akt1 (PDB ID: 1UNQ) [60] was selected as the template on the basis of good query coverage, sequence similarity (81%) and the highest resolution (0.98 Å). Clustal Omega [61] was used with the default parameters to retrieve the template-target sequence alignment, and the results were analysed via Jalview [62] which aided in the manual editing of the aligned sequence. Briefly, a total of 100 models were built using Modeller software version 9.12 [63]. The selection of the final model was based on the energy values of each model as indicated by the ERRAT score [64]. The energy of the selected model was further minimized using a MMFF94 force field [56] and validated via Ramachandran analysis [65], which evaluates each side chain conformation against an updated rotamer library. A homology model with a maximum percentage of residues or peptide bonds in the allowed regions of the Ramachandran plot was selected for a further docking study.

### Docking and Pose Analysis

To elucidate the ligand-receptor binding mode, and to obtain most probable binding conformations of the dataset for the GRIND analysis, 12 training set quinoline compounds were docked into a homology model of the Akt2 PH domain.

Compound selection for molecular docking was based on a comprehensive structural activity relationship (SAR) analysis as explained in the results and discussion section. Pre-processing of all the ligands was done with MOE version 2013.0802 [55]. The docking simulation was performed with the energy minimized conformation of the ligands using the GOLD suite v 5.2.2 [66]. To ensure the robustness of the docking solutions, 100 poses per ligand were generated and further evaluated by Gold fitness score.

To evaluate the Gold score fitness function, a decoy test set of 5200 compounds along with the selected 12 quinolines were re-docked in the binding pocket of the PH domain. The decoy dataset against subtype Akt2 was retrieved from the online decoy database DUD-E [67] and energy minimized via the MMFF94 force field [56]. To estimate the effectiveness of the docking solutions obtained, a simplest enrichment factor (EF) metric method [68] at a given percentage (1%, 2%, 5%) of the database was used, which is defined as:

EF=((tp/((tp+fp))) /TA)/N

Where tp and fp represent the number of true and false positives, TA is the total number of active compounds, and N is the total number of active and inactive compounds. The results generated by molecular docking were visualized using a receiver operator characteristic (ROC) curve where the true positive rate was plotted against the false positive rate. The area under the ROC curve was used as an evaluation metric.

A complete pose selection methodology is provided in the supporting information (S1 Fig). Briefly, to obtain the most probable binding conformations of selected quinoline analogues, the docking solutions were further analysed using an agglomerative hierarchical cluster analysis based on the RMSD of a common ligand scaffold (common scaffold clustering) [69]. The clustering the binding poses of selected Akt2 inhibitors resulted in a total of 24 clusters at a RMSD of 2.5 Å. Of these, only one cluster, which was designated as cluster I, accommodated all the docked ligands (12); however, another cluster that was designated as cluster II with 10/12 docked ligands was obtained. In order to remove any bias in the pose selection procedure, both clusters were included for further ligand-protein interaction studies. To further prioritize the clusters, the ligand-protein interaction patterns of both clusters were compared with SAR data as explained in the results and discussion section. Furthermore, molecular conformations in the selected cluster were used for the GRIND analysis, which was highly dependent on the 3D conformations of the molecules.

### Grid-Independent Molecular Descriptor (GRIND) Analysis

Selected molecular conformations of quinoline analogues were imported into Pentacle v 1.07 [70] along with their inhibitory potency log(1/IC_50_) values. Molecular Interaction Fields (MIFs) were calculated using different probes such as ‘DRY’ for hydrophobic interactions, ‘N1’ for hydrogen bond acceptors, ‘O’ for hydrogen bond donors and ‘TIP’ for steric hot spots within the molecule. Moreover, each probe was iteratively placed via a GRID to calculate the total energy as the sum of the hydrogen bond energies (Ehb), the Lennard-Jones energy (Elj) and the electrostatic energy (Eel):

$$E_{xyz}=\sum E_{hb}+\sum E_{lj}+\sum E_{el}$$

The structural characteristics of the dataset explained by the GRIND descriptors were evaluated with the AMANDA algorithm [71], which extracts the regions with the most relevant MIFs. The default grid space (0.5) and the energy cut-off values for probes implemented in software Pentacle v 1.07 [70] were used to discretize the MIFs. The nodes with energy below the cut-off value were discarded. Consistently large auto and cross correlation (CLACC) algorithms [70] in GRIND were used to encode the pre-filtered nodes, producing persistent variable sets whose values were directly represented in a correlogram plot. The final GRIND model was developed and validated using the leave-one-out method as explained in 2D-QSAR. Models with statistically significant *r*^*2*^, *q*^*2*^ and standard error values were further validated using an external test set of previously discovered quinoline inhibitors.

## Results and Discussion

### Hansch Analysis

A multiple linear regression analysis was performed using a set of 2D physicochemical descriptors to identify the most important physicochemical features for the high inhibitory potency of quinoline-type inhibitors of Akt2. The following equation is solely based on molar refractory (MR) and was derived using the contingency analysis tool in MOE to identify the most important descriptors (Eq 1):

$$\log\left(1|{IC}_{50}\right)=-3\cdot 52250+0.23173*\mathrm{MR}$$ (1)

$$n=89,r^{2}=0.56,q_{\left(LOO\right)}^{2}=0.54,\mathrm{RMSE}=0.40$$

**Fig 1:**
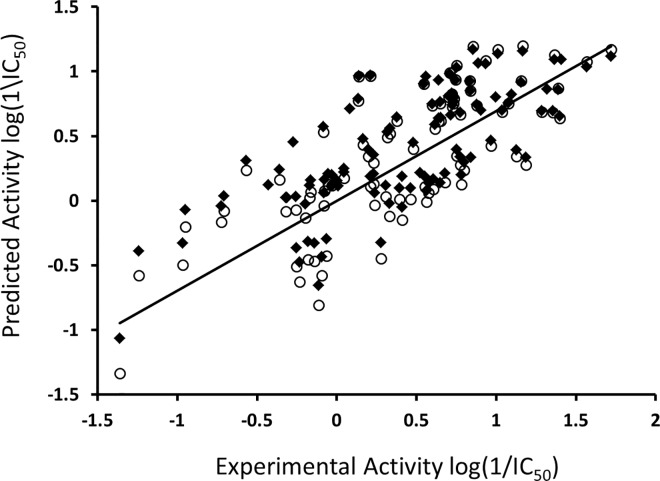
Experimental versus predicted inhibitory potency (log(1/IC_50_)) values of Akt2 inhibitors. The data points in square and circle represent training and test set compounds, respectively.

It is evident from the equation that MR is positively correlated with the inhibitory potency of quinoline derivatives against the Akt2 PH domain. However, no correlation has been identified between lipophilicity and the log(1/IC_50_) value for the dataset (data not shown). This indicates that overall polar interactions such as hydrogen bond formation have a positive impact on the IC_50_ values of quinoline derivatives against the target protein. So far, no reports that indicate that MR has an important effect on the high inhibitory potency of Akt2 inhibitors are available. Fig 1 shows a plot of experimental and predicted inhibitory potency log(1/IC_50_) values of the training and test set compounds. No outliers were observed in the training data (square), and all compounds were well-predicted with a residual value of less than one log unit and *q*^*2*^ and *r*^*2*^ values of 0.54 and 0.56, respectively.

The external test set was also used to further validate the final model, and all compounds in the test set (circle) were predicted with a difference of less than one log unit between the experimental and predicted inhibitory potency (log(1/IC_50_)) for Akt2 (Fig 1). This further demonstrated the good predictive ability of the selected QSAR model.

### Homology Modelling

**Fig 2:**
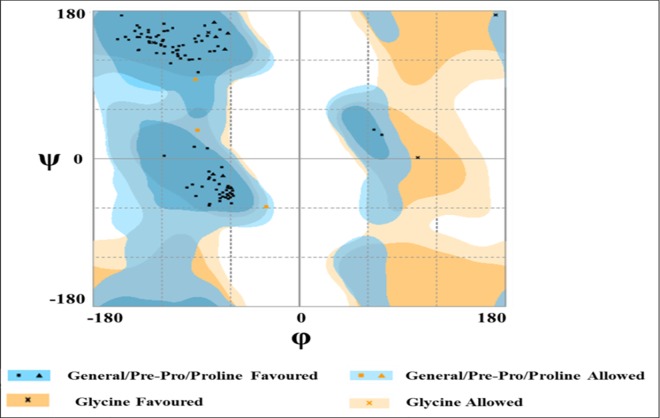
Ramachandran plot for Akt2 PH domain homology model. The plot shows 104 amino acid residues in the favoured region and three amino acid residues in allowed region. None of the amino acid residues lies in disallowed region.

Because a crystal structure of the Akt2 PH domain was unavailable, a homology model was constructed using the highly resolved crystal structure (0.98 Å) of the Akt1 PH domain (PDB ID: 1UNQ) as a template [59]. The template structure of the activated state showed the highest sequence similarity (81%) with the target protein. A Ramachandran plot (Fig 2) indicated that 97% of the residues lie in the favourable region and 3% in the allowed region; moreover, no residue was seen in the outlier region, which further strengthened the reliability of the final model.

### Docking and Pose Analysis

In order to shed light on the 3D interaction pattern and to obtain the most probable binding conformation of ligands for further GRIND studies, selected pyrido [2,3-d]-pyrimidine analogues were docked into an Akt2 homology model.

**Fig 3:**
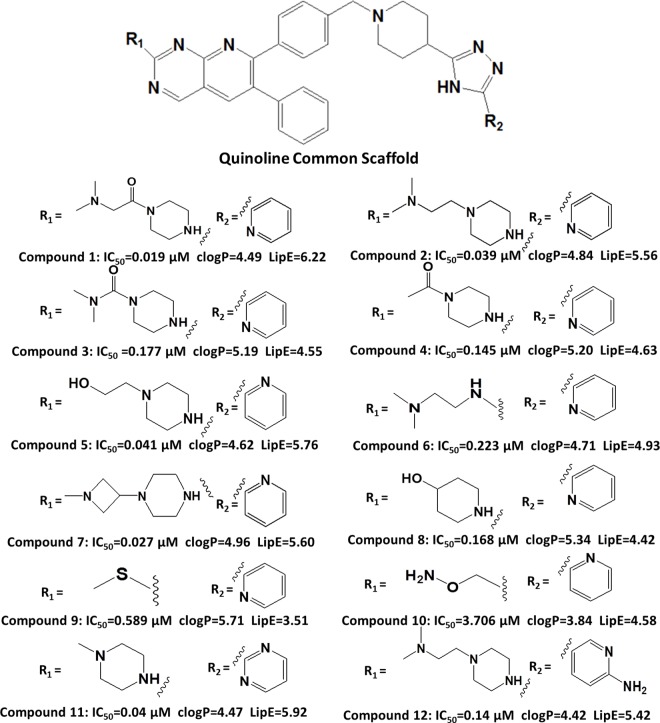
Selected pyrido [2, 3-d]-pyrimidine analogues used for molecular docking along with inhibitory potency (IC_50_ μM), clogp and LipE values.

Compounds selection (Fig 3) was solely based on the SAR data. Overall, the inhibitory potency values of the selected compounds varied from 0.019 μM to 3.706 μM. Compounds **1–6** were selected to highlight the importance of carbonyl, methyl and a terminal amino substituents on the piperazine ring of R1. For instance, a direct comparison of compounds **1** and **2** revealed that the presence of a carbonyl group adjacent to the piperazine ring at R1 increased the inhibitory potency of compound **1** (IC_50_(**1**): 0.019 μM) as compared to compound **2** (IC_50_(**2**): 0.039 μM). The two-fold greater inhibitory potency of compound **1** might be due to an additional hydrogen bond interaction of the carbonyl group at R1. This hypothesis was further strengthened because the removal of the piperazine moiety and the replacement of the carbonyl group with an amino group in compound **6** led to a two-order-of-magnitude decrease in the inhibitory potency of compound **6** compared to compound **1** (IC_50_(**1**): 0.019 μM, IC_50_(**6**): 0.223 μM). Additionally, the removal of one CH_2_ group adjacent to the carbonyl group at R1 (IC_50_ (**3**):0.177 μM) decreased the inhibitory potency of compound **3** by approximately one order of magnitude compared to compound **1**. This might indicate a contribution from lipophilic efficiency [72] towards inhibitory potency (LipE(**1)**: 6.22, LipE(**3)**: 4.55). Interestingly, no significant changes in the inhibitory potency of compounds **3** and **4** have been observed after removal of a terminal tertiary amino group at R1 in compound **4** (IC_50_(**3**): 0.177 μM, IC_50_(**4**): 0.145 μM). Similarly, the replacement of a terminal amino group in compound **2** with an OH group in compound **5** had a negligible impact on the inhibitory potency (IC_50_(**2**): 0.039 μM, IC_50_(**5**): 0.040 μM). Compounds **8**–**10** were selected to evaluate the impact of R1 groups other than a piperazine ring on the inhibitory potency against Akt2. Compounds **11** and **12** were selected to investigate the role of pyrimidine (**11**) or 3-pyridine (**12**) moieties at the R2 position in the ligand-protein interaction.

**Fig 4:**
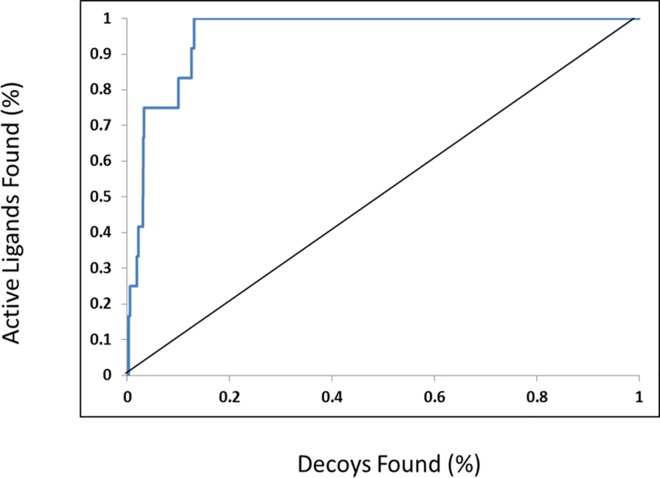
Comparison of the enrichments attained using the active quinoline ligands and DUD-E decoy sets over the Akt2 protein target. Enrichment is measured using the receiver operator characteristic (ROC) curves. The curve shows the fraction of selected decoys (x-axis) versus the fraction of active ligands (y-axis) ranked by their docking score.

Briefly, the binding site selected included an area of 15 Å near previously known interacting amino acid residues (Lys14, Arg15, Gly16, Glu17, Tyr18, Ile19, Lys20, Thr21, Arg23, Pro24, Arg25, Lys39, Pro51, Leu52, Asn53, Asn54, Phe55, Gln79, Ile84, Glu85, Arg86 and Phe88) defined in mutagenesis studies [23, 25]. The Gold Score was used as the fitness score to rank 100 poses per ligand. To evaluate the binding mode prediction, 12 active quinoline derivatives from the training set and 5200 decoy compounds retrieved from DUD-E [66] as the test set were re-docked within the Akt2 PH domain. The results were further analysed using the enrichment factor (EF) [68]. Respective EF values of 28.5, 19 and 21.3 at 1%, 2% and 5% from the dataset show that the docking methodology enriched the set of active compounds. The results were plotted with a ROC enrichment curve (Fig 4). The area under the enrichment curve showed that the discovery rate for active ligands was significantly higher than that for inactive ligands, which was helpful to differentiate the binding mode of active compounds within the binding pocket. These results supported the use of the docking model as a valuable tool for predicting the potential binding mode of selected quinoline compounds and further validated our Akt2 homology model.

A hierarchical cluster analysis based on the RMSD of the common scaffold of the docked ligands produced a total of 24 clusters at an RMSD of 2.5 Å. Only one cluster, which was designated as cluster I, contained all 12 docked ligands. We also identified another cluster designated as cluster II that contained 10 out of 12 docked ligands. The binding conformations of compounds **10** and **12** were missing in cluster II.

**Fig 5:**
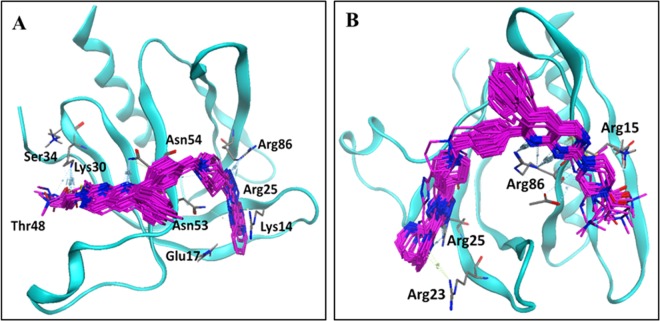
Docking solutions of Akt2 obtained from common scaffold cluster analysis. (A) Binding conformation of cluster I containing 12/12 docked ligands. (B) Binding conformation of cluster II containing 10/12 docked ligands.

Briefly, the binding solutions for cluster I showed that interactions with Lys14, Glu17, Arg25, Lys30, Ser34, Asn53, Asn54 and Arg86 amino acid residues were present at 2, 4 and 7 strands of beta sheets and Thr48 at the 1^st^ helical region of the binding cavity, as shown in Fig 5A. Of these residues, Lys30, Ser34 and Thr48 are mainly involved in hydrogen bonding with different substitutions at R1. The R2 group is surrounded by the Lys14, Glu17, Arg25, Asn53, and Arg86 amino acid residues while Asn54 shows hydrogen bond interactions with the quinoline scaffold. Binding solutions for cluster II showed interactions involving Arg15, Arg23, Arg25 and Arg86 amino acid residues in 1, 2, 4 and 7 strands of the beta sheets, as shown in Fig 5B. Of these, only Arg15 is involved in hydrogen bonding with R1, while Arg23 and Arg25 residues showed interactions with substitutions at R2, as shown in Fig 5B.

**Fig 6:**
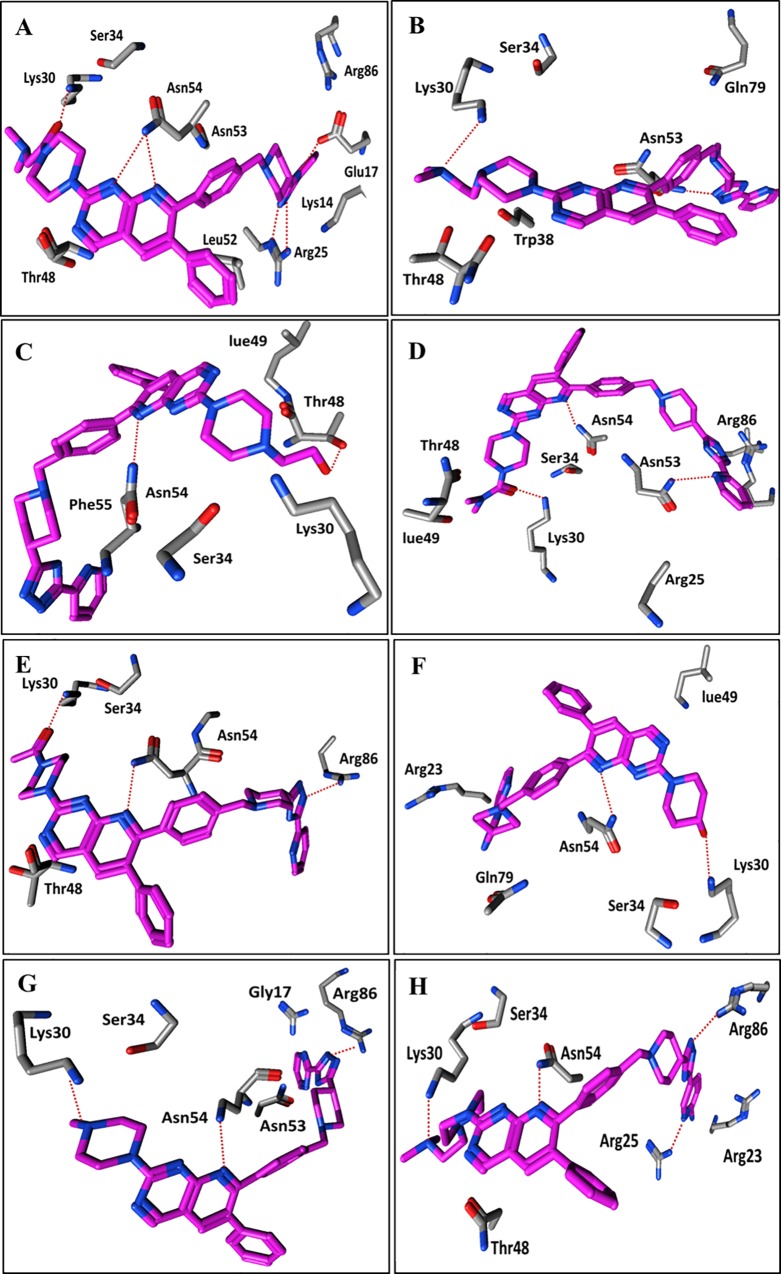
Interaction pattern of binding solutions in cluster I. (A & B): Ligand-protein interaction of compound **1** and **2**. (C, D & E): Interaction pattern of compounds **5, 3** and **4** within the binding cavity of PH domain of Akt2. (F, G & H): Binding poses of compound **8, 11** and **12** inside the PH domain of Akt2.

In the final docking solution of compound **1** (cluster I), the quinoline and triazole rings in the common scaffold showed respective hydrogen bond interactions with Asn54 and Arg25. However, carbonyl group at the R1 position forms a hydrogen bond with Lys30, as shown in Fig 6A. This is further supported by the finding of Meuillet [73], who demonstrated the interaction of Lys30 with sulfonamide type inhibitors against the PH domain of Akt. Moreover, a different study elucidated that the Lys30 and Lys389 pair is involved in cross-linking between the PH domain and the kinase domain of Akt, thus playing a crucial role in the inter-domain conformational changes during activation and ligand binding [74].

Additionally, ligand-protein interaction profiles of the docking solutions for compound **2** revealed that Lys30 forms a hydrogen bond with a terminal amino group at R1 (Fig 6B). However, replacement of the terminal amino group of compound **2** at the R1 position with an OH group in compound **5** resulted in a hydrogen bond interaction shift from Lys30 to Thr48 (Fig 6B & 6C) respectively, thus resulting in a negligible difference between the inhibitory potencies (IC_50_: 0.039 μM, IC_50_: 0.041 μM) of the two compounds. Interestingly, in both compounds **2** and **5,** the carbonyl group at R1 (Fig 6B & 6C) and C = O…NH-Lys30 interactions are absent, which further strengthens our hypothesis that the two-fold greater inhibitory potency of compound **1** compared to compounds **2** and **5** is might be due to the additional hydrogen bond interaction between Lys30 and the carbonyl group at R1. Interestingly, the carbonyl group at the R1 position in both compounds **3** and **4** also showed hydrogen bond interactions with Lys30, and the quinoline common scaffolds showed hydrogen bond interactions with Asn54 as in compound **1** (Fig 6A, 6D & 6E). However, we did not find any clear interaction for CH2 and terminal amino substitutions in the propanamide chain of R1 in compound **1**, but their absence in compound **3** and **4** resulted in an order of magnitude decrease in the inhibitory potency (IC_50_) values. This might be due to the highly lipophilic characteristics of compound **1** and potentially its high membrane permeability (LipE **1:** 6.22) compared to compounds **3** (LipE **3:** 4.55) and **4** (LipE **4:** 4.63), as shown in Fig 3. Similarly, replacement of the propanamide chain at R1 with **an** OH group results in a two-order-of-magnitude decrease in the inhibitory potency of compound **8** (IC_50_: 0.168 μM) compared to compound **1** (IC_50_: 0.019 μM). However, the OH group at R1 of compound **8** also forms a hydrogen bond with Lys30 (Fig 6F). As a consequence, a two order of magnitude decrease in the inhibitory potency of compound **8** compared to compound **1** (Fig 6A & 6F) could also be attributed to its low lipophilic efficiency (LipE(**1**): 6.22; LipE(**8**): 4.42). Thus, the propanamide chain at R1 might provide a basis for the high lipophilic efficiency in the membrane permeability for quinoline-type inhibitors against the Akt2 PH domain. A similar trend has been observed for compound **11,** in which the interaction with Lys30 was shifted to the piperazine ring in the absence of an entire propanamide chain at R1 (Fig 6F & 6G). Overall, a decreasing trend in the inhibitory potency and lipophilic efficiency of compounds **6**, **9** and **10** (IC_50_ (**6**/**9**/**10**): 0.22 μM, 0.59 μM, 3.71 μM) (LipE (**6**/**9**/**10**): 4.93, 3.51, 4.58) was observed in the absence of the piperazine ring and the replacement of propanamide chain with a primary amino, sulphide, and ether derivative at the R1 position, respectively. Moreover, a hydrogen bond interaction was observed between Lys30 and the primary amino group at R1 in compounds **6** and **10**, which further stresses the importance of the piperazine and propanamide chains for the high potency of the quinoline analogues. In general, the amino group of pyridine at the R2 position and the triazole ring of the common scaffold are surrounded by Arg25, Asn53, and Arg86 amino acid residues (Fig 6A–6H) in all docked ligands in cluster I.

**Fig 7:**
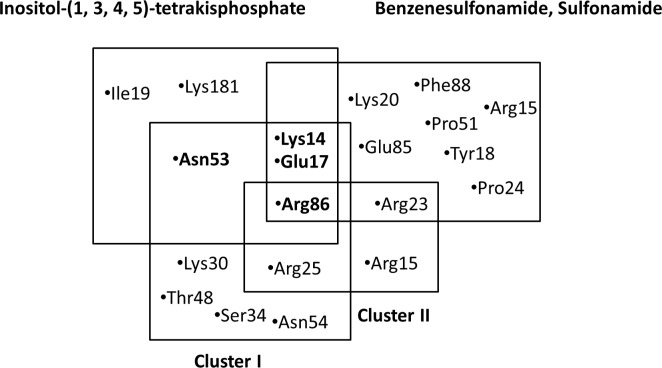
An overlap of interacting amino acid in cluster I & II with inositol-(1,3,4,5)-tetrakisphosphate, benzenesulfonamide and sulfonamide derivatives within Akt2 binding cavity.

Similarly, the R1 group in the binding conformations of cluster II showed hydrogen bond interactions with Arg15, whereas the analogous pyridine at R2 interacted with Arg25 and Arg86 (Fig 5B). Overall, no SAR pattern was observed for the binding solutions of quinoline analogues in cluster II. Therefore, to select the final cluster for further GRIND analysis, the main positioning and interacting amino acid residues of both clusters of quinoline analogues with those of previously known binding interactions of inositol-(1, 3, 4, 5)-tetrakisphosphate, benzenesulfonamide and sulfonamide derivatives [23, 25] with the Akt2 PH domain were compared. An overlap of Arg86 was observed between the quinoline analogues in the clusters, the sulfonamide and the inositol derivatives. Additionally, Lys14 and Glu17 showed an overlap between cluster I, the sulfonamide and the inositol derivatives. Asn53 only showed an overlap between cluster I and the inositol derivatives. Cluster II only showed an overlap of Arg23 with the sulfonamide derivatives as shown in Fig 7.

In conclusion, the ligand-protein interaction pattern identified in cluster I (maximum docked ligands cluster i.e. 12/12) was supported by the SAR data as well as by previous interaction data [23, 25] for the series. Therefore, cluster I was selected as the template for flexible alignment [75] of the rest of the quinoline dataset (S1 Table). The obtained ligand conformations were used to build a GRIND model for the entire dataset to investigate the 3D structural requirements of the ligands.

### Grid-Independent Molecular Descriptor (GRIND) Analysis

**Fig 8:**
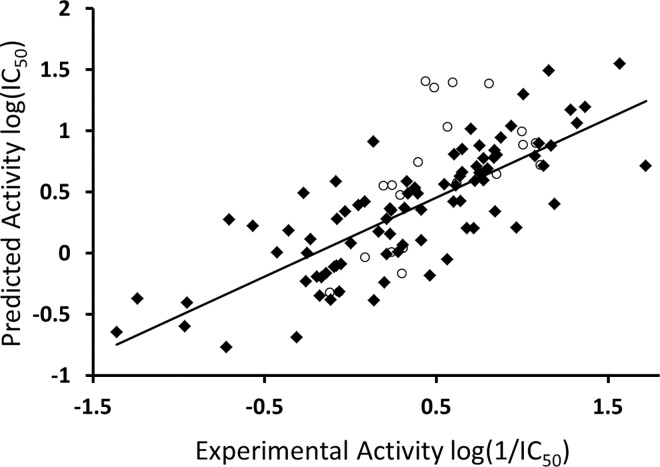
Experimental versus predicted inhibitory potency (log(1/IC_50_)) values of quinoline type inhibitors of Akt2. The data points in square and circle represent training and test set compounds respectively.

The molecular conformations of the data series obtained in the previous step, along with their Akt2 inhibitory potency (log(1/IC_50_)) values were used as inputs for the Pentacle v 1.07 software package [70] to develop a 3D-QSAR model using GRIND descriptors. A partial Least Square (PLS) analysis using the leave-one-out (LOO) cross-validation procedure [57] was carried out to correlate the inhibitory potency (log(1/IC_50_)) values with the 3D molecular structures. However, inconsistencies in some of the resultant variables produced a statistically inferior model. A fractional factorial design (FFD) variable selection algorithm [76] was applied to eliminate the inconsistencies in selected variables. The final model had satisfactory statistical parameters (*r*^*2*^ = 0.80, *q*^*2*^ = 0.63) and the standard error of prediction (RMSD) was 0.37. Fig 8 is representing a plot of actual versus predicted inhibitory potency (log(1/IC_50_)) values (S2 Table) obtained after the LOO cross validation of the training set. It shows that all compounds in the training set were predicted with a residual difference of less than one. Furthermore, evaluation of the training model by an external test set predicted inhibitory potency (log(1/IC_50_)) values of the test set data with a difference of less than one log unit between the experimental and predicted log(1/IC_50_) values (Fig 8; S2 Table). No false positive or false negative predictions in both training and test data further emphasize the robustness of the final GRIND model.

**Fig 9:**
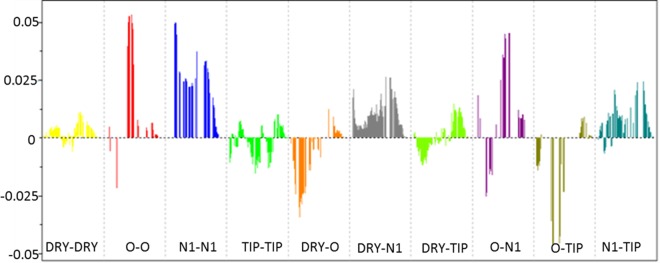
PLS coefficient profile of variables contributing positively (sharp positive peaks) and negatively (sharp negative peaks) towards inhibitory potency of quinoline type inhibitors of Akt2. The activity of the compounds predominantly increases with the increase in (O–O) and (N1–N1) variable value.

A PLS coefficient correlogram of the GRIND variables is shown in Fig 9 and depicts important 3D structural features that directly/inversely correlate with the inhibitory potencies of the compounds in the dataset. The PLS coefficient correlogram indicates that the O-O and N1-N1 variables are the primary positive contributors to the overall potency of the Akt2 inhibitors. However, O-TIP has a negative contribution towards the biological activities of compounds.

**Fig 10:**
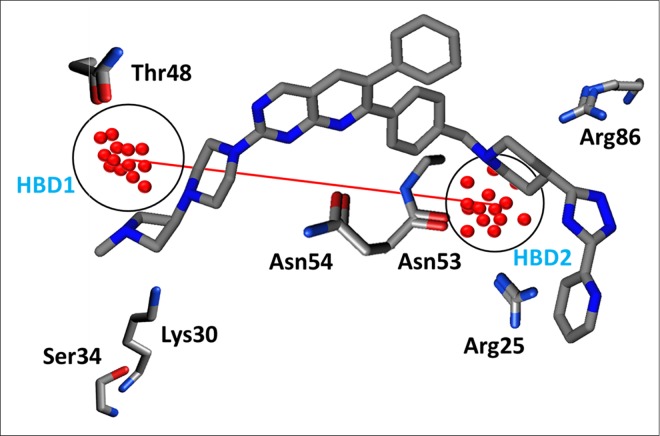
Two hydrogen bond donor HBD (O-O) hotspot regions at a distance of 15.2–15.6 Å apart within the molecule.

More explicitly, the O-O correlogram shows two hydrogen bond donor contours designated as HBD1 and HBD2 at a mutual distance of 15.2–15.6 Å within the molecule (Fig 10, Table 1) that represent the distance between the terminal amino groups at R1 and the piperidine rings at the common scaffold in the data series, as shown in Fig 10. Interestingly, this distance feature has been observed for the highly active compounds (log(1/IC_50_)<1.00) but it is absent in the least active (log(1/IC_50_)>1.00) compounds. Additionally, the backstage projection of the Akt2 PH domain homology model into identified hydrogen bond donor hotspots revealed the presence of complementary carbonyl groups in the Thr48 and Asn53 amino acid residues within the binding cavity, as shown in Fig 10. This further strengthened our docking outcomes and the validity of our pose selection criteria. A previously pharmacophore-based study on a benzene sulfonamide analogue revealed the presence of a single hydrogen bond donor (HBD) in most potent inhibitors of the Akt2 PH domain [23]. The difference in number of hydrogen bond donors in both studies might be because different chemical series have different hydrogen bond interaction patterns with the binding cavity of the Akt2 PH domain.

**Table 1:** Important molecular features along with their mutual distances for inhibitory potency values of quinoline compounds against Akt2.

Molecular Features	HBD2	HBA2	HY1
**HBD1**	15.2–15.6 Å	-	-
**HBA1**	16.8–17.2 Å	21.2–21.6 Å	23.6–24.0 Å
**HBA3**	7.2–7.6 Å	-	-

**Fig 11:**
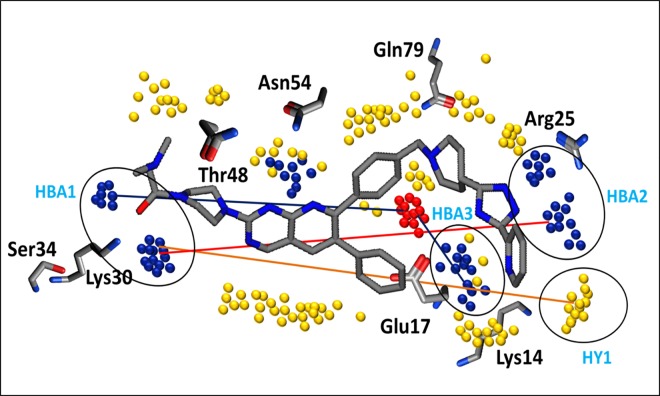
Important hotspots regions for the high inhibitory potency of Akt2 inhibitors. Red distance line: Representing a distance of 21.2–21.6 Å between two hydrogen bond acceptors HBA1 and HBA2 (blue hotspots) within the molecule. Orange distance line: Representing a distance of 23.6–24.0 Å between hydrophobic HY contours (yellow hotspots) from hydrogen bond acceptor (HBA1) feature. Blue distance line: Representing hydrogen bond donor HBD2 (red hotspots) at a distance of 7.2–7.6 and 16.8–17.2 Å from hydrogen bond acceptor HBA3 and HBA1, respectively.

Similarly, the N1-N1 variables in Fig 9 show a positive impact of two hydrogen bond acceptors at a mutual distance of 21.2–21.6 Å towards the inhibitory potency of the quinoline analogues, indicating the distance between the carbonyl group at R1 (HBA1) and the triazole at R2 position (HBA2) as shown by the red distance line in Fig 11. Interestingly, the two hydrogen bond acceptor regions identified (blue hotspots) and their mutual distances are the complementary NH groups of the Lys30 and Arg25 amino acid residues. This was confirmed by our docking results, which demonstrated the importance of Lys30 and Arg25 for the interaction with the binding cavity of the Akt2 PH domain. These outcomes are also in accord with another pharmacophore-based study on the isoquinoline analogues of Akt2 inhibitors that demonstrated that two hydrogen bond acceptors (HBA) and one hydrogen bond donor (HBD) were crucial for the high inhibitory potency (IC_50_) of isoquinoline analogues against the PH domain of the target protein [77]. However, a recent structure-based study on the kinase domain of Akt1 indicated the significance of one hydrogen bond acceptor and two hydrophobic features in the ligand-protein interaction profiles of diphenylmethylamine derivatives [78]. Similarly, another study on a diverse dataset of compounds revealed the presence of two hydrogen bond acceptors and two hydrophobic features interacting in highly active PH domain inhibitors of Akt1 [25]. The difference in the number and pattern of hydrogen bond acceptor regions in these different studies might be due to the difference in the chemical scaffolds of the training set that might interact differently. Additionally in our study, a hydrogen bond acceptor (HBA1) at a distance (orange distance line) of 23.6–24.0 Å from a hydrophobic feature (DRY-N1) within the molecules positively contributes to the overall inhibitory potency of the quinoline analogues, as shown in Fig 11 & Table 1. Interestingly, the hydrogen bond acceptor region (HBA1) in the molecule is the carbonyl group at R1 that is complementary to the NH group of Ly30 and the hydrophobic region (HY) (yellow hotspots) is a pyridine substituent at R2 that is complementary to the aliphatic chain of Lys14 (Fig 11). This is in accord with our SAR and docking outcomes regarding the role of carbonyl group at the R1 position that confer high inhibitory potency on the quinoline analogues against the Akt2 PH domain. This is also in accord with two different 3D-QSAR and pharmacophore studies that indicated that one hydrophobic, one aromatic and one hydrogen bond acceptor affected the inhibitory potency of pyrrolo-pyrimidine analogues against Akt2 [79, 80].

The presence of one hydrogen bond donor and one steric hotspot indicate a molecular boundary (O-TIP) at a mutual distance of 13.20–13.60 Å that has a negative effect on the inhibitory potency against Akt2 inhibitors. This particular pattern was observed in 19% of compounds while the rest of the dataset showed arbitrary behaviour which indicates the importance of a single hydrogen bond donor at a certain distance from the steric hotspot region within the cavity of Akt2 PH domain. Furthermore, the O-N1 pair of probes in the PLS coefficient correlogram (Fig 9) means that the hydrogen bond donor (HBD2) and the hydrogen bond acceptor (HBA3) feature at a mutual distance (blue line) of 7.2–7.6 Å have a negative impact on the overall inhibitory potency against Akt2; however, similar features (HBD2 and HBA1) that are present at a longer distance (blue distance line) range of 16.8–17.2 Å have a positive effect on the biological activity of the quinoline analogues against the Akt2 PH domain. HBD2 and HBA3 represent the NH of the triazole group in the common scaffold of the series and the amino group of piperidine, respectively. Moreover, the carbonyl group at R1 and the pyridine ring of the common scaffold respectively encode HBA1 and HBD2 at a greater distance (Fig 11). The hydrogen bond acceptor HBA1 (blue hotspots) and donor HBD2 (red hotspots) at a distance of 16.8–17.2 Å are complementary to an amino group of Lys30 and a carbonyl group of Glu17. Thus, Lys30 represents the most important interacting region in the Akt PH domain. The distances of the other amino acid residues such as Glu17, Arg25 and Lys14 that are complementary to identified pharmacophoric features have been calculated from Lys30 and in turn complement the carbonyl group at R1.

Previously, Vyas and colleagues developed CoMFA and CoMSIA models using carboxamide, pyrimidine and phenylpurine series of compounds against the Akt2 catalytic domain. Steric and electrostatic features have been shown to play an important role in the high inhibitory potency against the Akt2 catalytic domain [81]. In another study, a pharmacophore model of pyrimidine analogues indicated that two hydrophobic features, one hydrogen bond donor and one hydrogen bond acceptor were important in the overall activity of the compounds against the Akt2catalytic domain [80]. Overall, our study provided a deeper insight into ligand binding at the Akt2 PH domain by mapping the mutual distances of important pharmacophoric features (two hydrogen bond donors, three hydrogen bond acceptors and one hydrophobic feature) as well as the complementary distances of the interacting amino acid residues (Lys30, Arg25, Glu17 and Lys14) in the Akt2 PH domain, as shown in Table 1. Moreover, targeting the Akt PH domain might allow isoform selectivity and thus could help reduce off-target toxicity during cancer chemotherapeutic treatments. Based on our current findings, we suggest that a more potent inhibitor against the Akt2 PH domain might be obtained by (i) increasing the hydrogen bond acceptor strength at R1; (ii) increasing the length of the propanamide chain at R1 to aid in achieving a high lipophilic efficiency or membrane permeability; (iii) maintaining a hydrogen bond acceptor (R1) distance of 16.8–17.2 Å, 21.2–21.6 Å and 23.6–24.0 Å from a hydrogen bond donor, another hydrogen bond acceptor and a hydrophobic group, respectively; and (iv) avoiding a hydrogen bond acceptor and donor distance of ≤ 7.6.

### External validation

**Fig 12:**
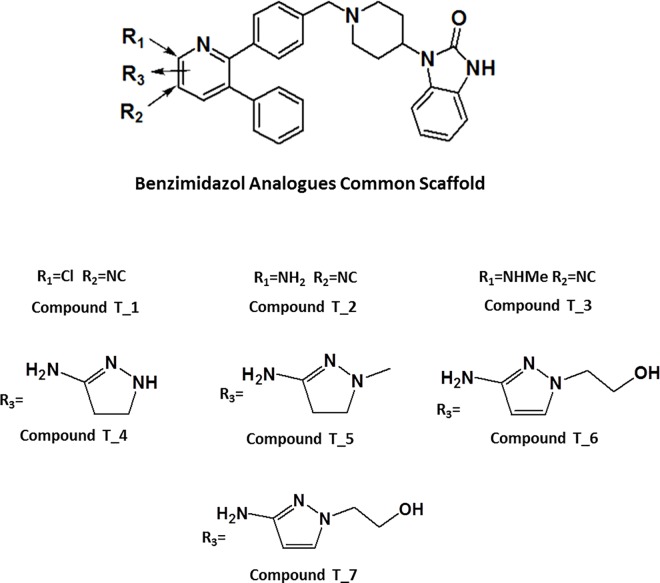
Selected 3-[1-[[4-(3-phenyl-2-pyridyl)phenyl]methyl]-4-piperidyl]-1H-benzimidazol-2-one analogues used for external validation.

In order to further elucidate the robustness of the final QSAR and GRIND models, a previously published external test set for benzimidazol-2-one was used for model evaluation [50] (Fig 12).

The biological activity (IC_50_) values of the test data against the Akt2 PH domain varied from 0.556–8.141 μM, and compound **T_5** was a highly active (IC_50_ = 0.556 μM) analogue, as shown in Table 2.

**Table 2:** Selected 3-[1-[[4-(3-phenyl-2-pyridyl)phenyl]methyl]-4-piperidyl]-1H-benzimidazol-2-one analogues used for external validation of 2D-QSAR and GRIND model along with experimental and predicted inhibitory potency (IC_50_ μM) values.

Compound	Experimental Biological Activity IC_50_ (μM) ± SE	log(1/IC_50_)	Predicted log(1/IC_50_)		logP(o/w)
			2D-QSAR	GRIND (3D-QSAR)	
**T_1**	7.356±5104	-0.867	-0.044	-0.102	6.379
**T_2**	2.062±1021	-0.314	-0.103	0.115	5.115
**T_3**	8.141±2584	-0.911	0.013	0.063	5.434
**T_4**	7.356±5104	-0.866	-0.410	0.099	5.892
**T_5**	0.556±26	0.255	0.065	0.061	5.505
**T_6**	1.228±26	-0.089	0.220	0.210	4.811
**T_7**	0.839±53	0.076	0.579	0.523	5.077

All inhibitory potency (IC_50_) values were expressed as the average of at least two determinations ± standard deviation. The biological activity values of all compounds in the external test set were predicted by our 2D-QSAR and GRIND models. All compounds in the test set were predicted with a difference of less than one log unit between the actual and predicted biological activity (Table 2).

Overall, in the present study, the binding hypotheses against the Akt2 PH domain derived from different models were complementary with each other as well as with the available mutagenesis data, thus indicating the fitness of our models. Moreover, the 2D-QSAR and GRIND models we developed also predicted the inhibitory potency of an external test set (Table 2) with reasonable accuracy, which further indicates the robustness of our models. In a follow-up study, further generalization of the fact will be elucidated by designing and pharmacological testing of new libraries of a diverse dataset of inhibitors against the Akt2 PH domain.

## Conclusion

In this study, we elucidated the binding hypothesis and 3D structural requirements for the selective inhibition of quinoline analogues in the binding pocket of the Akt2 PH domain. Although docking studies using homology models are always prone to error, our docking results augmented SAR, 2D-QSAR and Grid-Independent Molecular Descriptor (GRIND) analyses as well as the available experimental data. A Hansch analysis indicated the positive effects of hydrogen bond formation on the inhibitory potency against the target protein. Our GRIND model indicated the presence of two hydrogen bond acceptors, two hydrogen bond donors and one hydrophobic region at a certain distance in quinoline analogues that are highly potent against the Akt2 PH domain. A molecular docking study indicated the role of the Glu17, Arg25, Lys30, Asn52 and Arg86 residues in the interaction of the quinoline analogues in the binding region of the Akt2 PH domain. Notably, the Lys30 residue was the most important interaction point in the binding pocket complementary to the carbonyl group at R, which might act as an anchor point for mapping the distances of important pharmacophoric features identified by the GRIND model, thus confirming its contribution to the high inhibitory potency against the Akt2 PH domain. The application of a SAR-guided pose analysis to develop 3D-GRIND models to retrieve the binding hypothesis as well as the 3D structural requirements for inhibitors against the Akt2 PH domain could pave the way towards the design of antagonists specifically directed against the other isoforms of Akt (Akt1-3), and potentially guide the development of cancer-specific chemotherapeutic treatments with limited side effects.

## Supporting Information

S1 Fig Brief overview of pose selection methodology. (DOCX)

S1 Table Dataset of Akt2 inhibitors consist of quinoline type inhibitors of PH domain of Akt2 along with biological activity values (IC_50_ μM). (DOCX)

S2 Table Experimental and predicted biological activity values (log(1/IC_50_)) of training and test sets obtained after Leave-One-Out (LOO) cross validation. (DOCX)
