# Supplementary figures and images for: A 2D-QSAR and Grid-Independent Molecular Descriptor (GRIND) Analysis of Quinoline-Type Inhibitors of Akt2: Exploration of the Binding Mode in the Pleckstrin Homology (PH) Domain
Source: PLoS One. 2016 Dec 30;11(12):e0168806. doi: 10.1371/journal.pone.0168806 (PMC5201309; doi:10.1371/journal.pone.0168806)

**S1 Fig:** Brief overview of pose selection methodology.


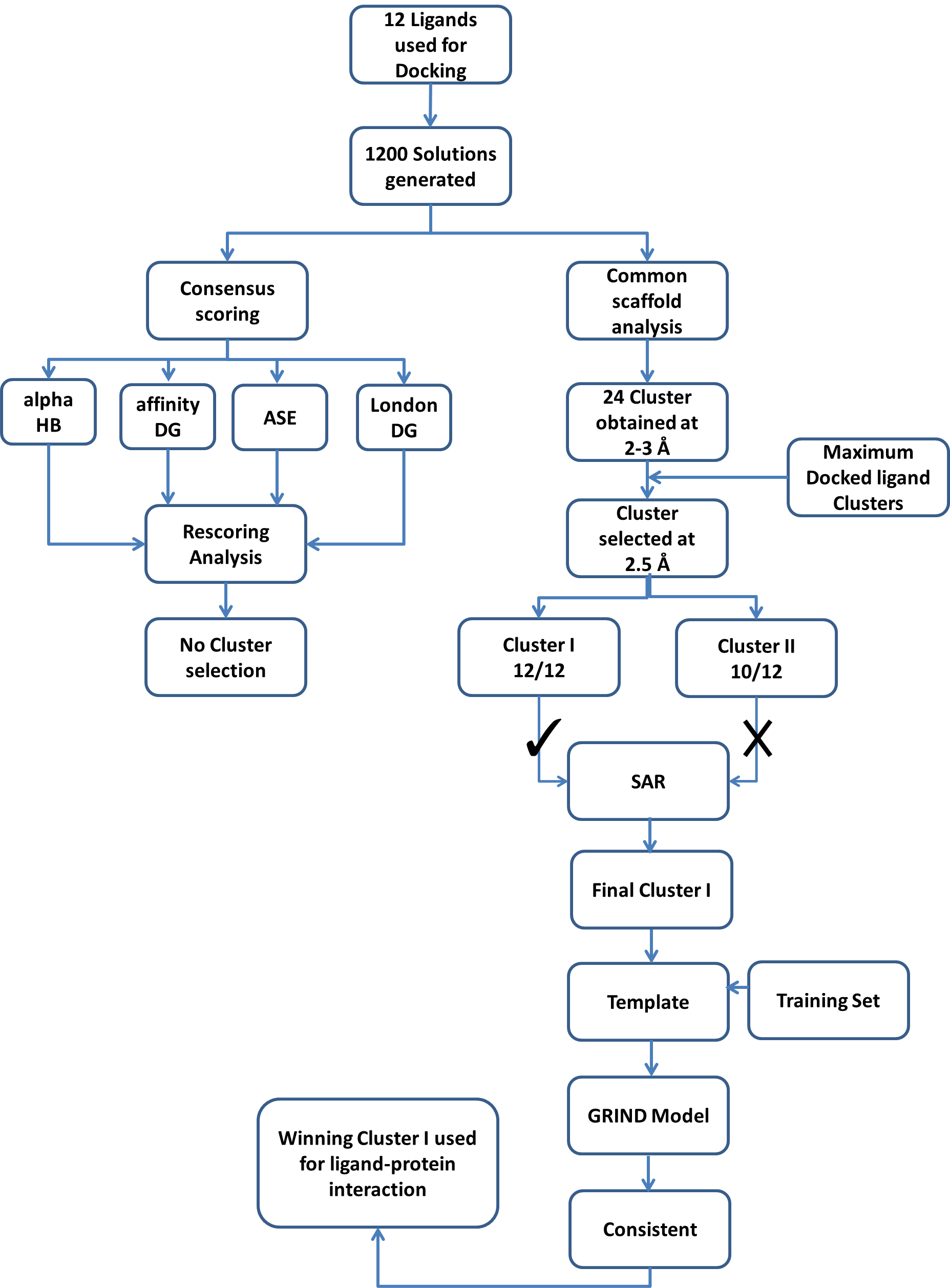

Supplement: S1 Fig — (DOCX) [file pone.0168806.s001.docx]
