# Supplementary material for: A 2D-QSAR and Grid-Independent Molecular Descriptor (GRIND) Analysis of Quinoline-Type Inhibitors of Akt2: Exploration of the Binding Mode in the Pleckstrin Homology (PH) Domain
Source: PLoS One. 2016 Dec 30;11(12):e0168806. doi: 10.1371/journal.pone.0168806 (PMC5201309; doi:10.1371/journal.pone.0168806)
Supplement: S1 Table — (DOCX) [file pone.0168806.s002.docx]

**S1 Table:** Dataset of Akt2 inhibitors consist of quinoline type inhibitors of PH domain of Akt2 along with biological activity values (IC_50_ µM).

| Quinolines_A | | | | | | | | | | | | | | | | | Quinolines_B | | | | | | | | | | | | | | | | | | | | |
| --- | --- | --- | --- | --- | --- | --- | --- | --- | --- | --- | --- | --- | --- | --- | --- | --- | --- | --- | --- | --- | --- | --- | --- | --- | --- | --- | --- | --- | --- | --- | --- | --- | --- | --- | --- | --- | --- |
|  | | | | | | | | | | | | | | | | |  | | | | | | | | | | | | | | | | | | | | |
| **#** | **scaffold** | | | | | **R_1_** | | | | | | | **R_2_** | | | | | | | | | **R_3_** | | | | | **IC_50_ (µM)** | **Molar refractivity** | | | | | | **LogP**  **(o/w)** | | | **ID** |
| 1 | (A) | | | | |  | | | | | | |  | | | | | | | | | - | | | | | 0.040 | 18.08313 | | | | | | 4.1191 | | | ph_28 |
| 2 | (A) | | | | |  | | | | | | |  | | | | | | | | | - | | | | | 0.084 | 18.51378 | | | | | | 4.179 | | | ph_29 |
| 3 | (A) | | | | |  | | | | | | |  | | | | | | | | | - | | | | | 0.188 | 18.41212 | | | | | | 3.452 | | | ph_30 |
| 4 | (A) | | | | |  | | | | | | |  | | | | | | | | | - | | | | | 0.185 | 18.5128 | | | | | | 4.19 | | | ph_31 |
| 5 | (A) | | | | |  | | | | | | |  | | | | | | | | | - | | | | | 0.080 | 18.78925 | | | | | | 3.4251 | | | ph_32 |
| 6 | (A) | | | | |  | | | | | | |  | | | | | | | | | - | | | | | 0.180 | 19.22007 | | | | | | 3.485 | | | ph_33 |
| 7 | (A) | | | | |  | | | | | | |  | | | | | | | | | - | | | | | 0.284 | 19.08734 | | | | | | 2.758 | | | ph_34 |
| 8 | (A) | | | | |  | | | | | | |  | | | | | | | | | - | | | | | 0.146 | 19.18789 | | | | | | 3.496 | | | ph_35 |
| 9 | (A) | | | | |  | | | | | | |  | | | | | | | | | - | | | | | 0.130 | 19.7697 | | | | | | 3.9961 | | | ph_36 |
| 10 | (A) | | | | |  | | | | | | |  | | | | | | | | | - | | | | | 0.098 | 20.10059 | | | | | | 3.329 | | | ph_38 |
| 11 | (A) | | | | |  | | | | | | |  | | | | | | | | | - | | | | | 0.068 | 20.2002 | | | | | | 4.067 | | | ph_39 |
| 12 | (A) | | | | |  | | | | | | |  | | | | | | | | | - | | | | | 0.899 | 16.32389 | | | | | | 5.0060 | | | ph_49 |
| 13 | (A) | | | | |  | | | | | | |  | | | | | | | | | - | | | | | 0.388 | 15.09404 | | | | | | 5.356 | | | ph_50 |
| 14 | (A) | | | | |  | | | | | | |  | | | | | | | | | - | | | | | 0.462 | 17.63606 | | | | | | 6.3819 | | | ph_51 |
| 15 | (A) | | | | |  | | | | | | |  | | | | | | | | | - | | | | | 0.229 | 17.99001 | | | | | | 6.2480 | | | ph_52 |
| 16 | (A) | | | | |  | | | | | | |  | | | | | | | | | - | | | | | 0.243 | 17.76716 | | | | | | 4.8400 | | | ph_53 |
| 17 | (A) | | | | |  | | | | | | |  | | | | | | | | | - | | | | | 0.140 | 20.19913 | | | | | | 4.056 | | | ph_37 |
| 18 | (A) | | | | |  | | | | | | |  | | | | | | | | | - | | | | | 0.168 | 18.18741 | | | | | | 5.2330 | | | ph_54 |
| 19 | (A) | | | | |  | | | | | | |  | | | | | | | | | - | | | | | 0.184 | 18.65214 | | | | | | 5.7640 | | | ph_55 |
| 20 | (A) | | | | |  | | | | | | |  | | | | | | | | | - | | | | | 0.101 | 18.69916 | | | | | | 5.4550 | | | ph_56 |
| 21 | (A) | | | | |  | | | | | | |  | | | | | | | | | - | | | | | 0.044 | 18.25666 | | | | | | 4.9930 | | | ph_57 |
| 22 | (A) | | | | |  | | | | | | |  | | | | | | | | | - | | | | | 0.125 | 18.25666 | | | | | | 4.9930 | | | ph_58 |
| 23 | (A) | | | | |  | | | | | | |  | | | | | | | | | - | | | | | 0.052 | 18.2652 | | | | | | 4.7990 | | | ph_59 |
| 24 | (A) | | | | |  | | | | | | |  | | | | | | | | | - | | | | | 0.199 | 18.70285 | | | | | | 5.4550 | | | ph_60 |
| 25 | (A) | | | | |  | | | | | | |  | | | | | | | | | - | | | | | 0.223 | 18.0049 | | | | | | 4.8830 | | | ph_61 |
| 26 | (A) | | | | |  | | | | | | |  | | | | | | | | | - | | | | | 0.421 | 18.00908 | | | | | | 4.0350 | | | ph_62 |
| 27 | (A) | | | | |  | | | | | | |  | | | | | | | | | - | | | | | 0.070 | 19.18402 | | | | | | 5.8040 | | | ph_63 |
| 28 | (A) | | | | |  | | | | | | |  | | | | | | | | | - | | | | | 0.684 | 17.29556 | | | | | | 4.1760 | | | ph_64 |
| 29 | (A) | | | | | **** | | | | | | |  | | | | | | | | | - | | | | | 0.145 | 18.88614 | | | | | | 4.3720 | | | ph_65 |
| 30 | (A) | | | | |  | | | | | | |  | | | | | | | | | - | | | | | 0.196 | 19.41308 | | | | | | 3.4240 | | | ph_66 |
| 31 | (A) | | | | |  | | | | | | |  | | | | | | | | | - | | | | | 0.177 | 19.6346 | | | | | | 4.2130 | | | ph_67 |
| 32 | (A) | | | | |  | | | | | | |  | | | | | | | | | - | | | | | 0.019 | 20.10537 | | | | | | 4.1230 | | | ph_68 |
| 33 | (A) | | | | |  | | | | | | |  | | | | | | | | | - | | | | | 0.735 | 18.58698 | | | | | | 3.8420 | | | ph_69 |
| 34 | (A) | | | | |  | | | | | | |  | | | | | | | | | - | | | | | 0.041 | 18.97058 | | | | | | 4.1050 | | | ph_70 |
| 35 | (A) | | | | |  | | | | | | |  | | | | | | | | | - | | | | | 0.027 | 19.73545 | | | | | | 4.4450 | | | ph_72 |
| 36 | (A) | | | | |  | | | | | | |  | | | | | | | | | - | | | | | 0.039 | 19.95094 | | | | | | 4.6760 | | | ph_21 |
| 37 | (A) | | | | |  | | | | | | |  | | | | | | | | | - | | | | | 1.299 | 12.6 | | | | | | 3.458 | | | ph_83 |
| 38 | (A) | | | | |  | | | | | | |  | | | | | | | | | - | | | | | 8.923 | 14.89716 | | | | | | 4.443 | | | ph_84 |
| 39 | (A) | | | | |  | | | | | | |  | | | | | | | | | - | | | | | 1.242 | 13.46508 | | | | | | 4.171 | | | ph_85 |
| 40 | (A) | | | | |  | | | | | | |  | | | | | | | | | - | | | | | 1.709 | 13.28369 | | | | | | 3.994 | | | ph_86 |
| 41 | (A) | | | | |  | | | | | | |  | | | | | | | | | - | | | | | 1.381 | 13.89409 | | | | | | 5.123 | | | ph_87 |
| 42 | (A) | | | | |  | | | | | | |  | | | | | | | | | - | | | | | 1.16 | 14.04704 | | | | | | 5.242 | | | ph_88 |
| 43 | (A) | | | | |  | | | | | | |  | | | | | | | | | - | | | | | 1.514 | 13.94035 | | | | | | 3.82 | | | ph_89 |
| 44 | (A) | | | | |  | | | | | | |  | | | | | | | | | - | | | | | 17.47 | 13.46508 | | | | | | 5.069 | | | ph_90 |
| 45 | (A) | | | | |  | | | | | | |  | | | | | | | | | - | | | | | 1.792 | 13.73266 | | | | | | 4.326 | | | ph_91 |
| 46 | (A) | | | | |  | | | | | | |  | | | | | | | | | - | | | | | 0.494 | 15.77562 | | | | | | 5.988 | | | ph_92 |
| 47 | (A) | | | | |  | | | | | | |  | | | | | | | | | - | | | | | 0.529 | 13.97062 | | | | | | 4.915 | | | ph_93 |
| 48 | (A) | | | | |  | | | | | | |  | | | | | | | | | - | | | | | 9.259 | 13.77832 | | | | | | 4.115 | | | ph_95 |
| 49 | (A) | | | | |  | | | | | | |  | | | | | | | | | - | | | | | 5.296 | 15.03822 | | | | | | 4.758 | | | ph_96 |
| 50 | (A) | | | | |  | | | | | | |  | | | | | | | | | - | | | | | 1.572 | 15.15437 | | | | | | 5.572 | | | ph_97 |
| 51 | (A) | | | | |  | | | | | | |  | | | | | | | | | - | | | | | 1.131 | 16.13418 | | | | | | 4.893 | | | ph_98 |
| 52 | (A) | | | | |  | | | | | | |  | | | | | | | | | - | | | | | 0.635 | 16.95525 | | | | | | 6.415 | | | ph_99 |
| 53 | (A) | | | | |  | | | | | | |  | | | | | | | | | - | | | | | 2.295 | 16.27466 | | | | | | 5.3150 | | | ph_101 |
| 54 | (A) | | | | |  | | | | | | |  | | | | | | | | | - | | | | | 0.586 | 16.14828 | | | | | | 4.6860 | | | ph_102 |
| 55 | (A) | | | | |  | | | | | | |  | | | | | | | | | - | | | | | 3.706 | 16.55119 | | | | | | 4.1720 | | | ph_103 |
| 56 | (A) | | | | |  | | | | | | |  | | | | | | | | | - | | | | | 0.589 | 16.77627 | | | | | | 5.9510 | | | ph_100 |
| 57 | (A) | | | | | - | | | | | | |  | | | | | | | | |  | | | | | 1.811 | 15.39011 | | | | | | 5.511 | | | ph_81 |
| 58 | (A) | | | | | - | | | | | | |  | | | | | | | | |  | | | | | 0.468 | 15.20386 | | | | | | 3.3135 | | | ph_82 |
| 59 | (A) | | | | |  | | | | | | |  | | | | | | | | |  | | | | | 0.577 | 15.52907 | | | | | | 3.849 | | | ph_129 |
| 61 | (A) | | | | |  | | | | | | |  | | | | | | | | |  | | | | | 0.274 | 15.61874 | | | | | | 3.485 | | | ph_130 |
| 62 | (A) | | | | | - | | | | | | |  | | | | | | | | |  | | | | | 5.084 | 15.36726 | | | | | | 3.804 | | | ph_131 |
| 63 | (A) | | | | | - | | | | | | |  | | | | | | | | |  | | | | | 0.344 | 15.69832 | | | | | | 4.129 | | | ph_132 |
| 64 | (A) | | | | | - | | | | | | |  | | | | | | | | |  | | | | | 1.198 | 15.94475 | | | | | | 4.267 | | | ph_134 |
| 65 | (B) | | | | | - | | | | | | | - | | | | | | | | | - | | | | | 2.057 | 15.34394 | | | | | | 6.2575 | | | ph_1 |
| 66 | (B) | | | | |  | | | | | | | - | | | | | | | | | - | | | | | 0.21 | 16.19917 | | | | | | 5.7135 | | | ph_2 |
| 67 | (B) | | | | | - | | | | | | | - | | | | | | | | |  | | | | | 0.281 | 16.0788 | | | | | | 5.9715 | | | ph_75 |
| 68 | (B) | | | | | - | | | | | | |  | | | | | | | | | - | | | | | 0.388 | 16.0788 | | | | | | 5.9715 | | | ph_76 |
| 69 | (B) | | | | | - | | | | | | | - | | | | | | | | |  | | | | | 0.065 | 16.71932 | | | | | | 5.6605 | | | ph_77 |
| 70 | (B) | | | | | - | | | | | | | - | | | | | | | | |  | | | | | 0.617 | 19.32423 | | | | | | 6.1525 | | | ph_79 |
| 71 | (B) | | | | | - | | | | | | |  | | | | | | | | | - | | | | | 0.276 | 19.32423 | | | | | | 6.1525 | | | ph_80 |
| 72 | (B) | | | | | - | | | | | | |  | | | | | | | | | - | | | | | 0.144 | 16.71932 | | | | | | 5.6605 | | | ph_149 |
| 73 | (B) | | | | | - | | | | | | | - | | | | | | | | |  | | | | | 1.877 | 17.1766 | | | | | | 5.2735 | | | ph_150 |
| 74 | (B) | | | | | - | | | | | | |  | | | | | | | | | - | | | | | 0.332 | 17.1766 | | | | | | 5.2735 | | | ph_151 |
| 75 | (B) | | | | |  | | | | | | | - | | | | | | | | | - | | | | | 0.3 | 16.19917 | | | | | | 6.3605 | | | ph_152 |
| 76 | (B) | | | | |  | | | | | | | - | | | | | | | | | - | | | | | 0.9 | 16.19917 | | | | | | 5.7135 | | | ph_154 |
| 77 | (B) | | | | | - | | | | | | | - | | | | | | | | | - | | | | | 2.09 | 15.34394 | | | | | | 6.257 | | | ph_136 |
| **Quinolines_C** | | | | | | | | | | | | | | | | | | | | | | | | | | | **Quinolines_D** | | | | | | | | | | |
|  | | | | | | | | | | | | | | | | | | |  | | | | | | | | | | | | | | | | | | |
| **#** | | | | | **scaffold** | | | | **R_1_** | | | **R_2_** | | | | | | | | **R_3_** | | | **IC_50_ (µM)** | | | **Molar refractivity** | | | | | | **LogP(o/w)** | | | | **ID** | |
| 78 | | | | | (C) | | | | - | | |  | | | | | | | | - | | | 0.168 | | | 16.7207 | | | | | | 4.69106 | | | | ph_109 | |
| 79 | | | | | (C) | | | | - | | |  | | | | | | | | - | | | 0.108 | | | 17.27438 | | | | | | 5.48806 | | | | ph_110 | |
| 80 | | | | | (C) | | | | - | | |  | | | | | | | | - | | | 1.213 | | | 17.68073 | | | | | | 3.57806 | | | | ph_111 | |
| 81 | | | | | (C) | | | | - | | |  | | | | | | | | - | | | 0.176 | | | 16.97583 | | | | | | 4.96406 | | | | ph_113 | |
| 82 | | | | | (C) | | | | - | | |  | | | | | | | | - | | | 0.483 | | | 17.51803 | | | | | | 5.39206 | | | | ph_114 | |
| 83 | | | | | (C) | | | | - | | |  | | | | | | | | - | | | 0.251 | | | 18.44986 | | | | | | 3.75306 | | | | ph_116 | |
| 84 | | | | | (C) | | | | - | | |  | | | | | | | | - | | | 0.075 | | | 16.96202 | | | | | | 3.70553 | | | | ph_117 | |
| 85 | | | | | (D) | | | |  | | | - | | | | | | | | - | | | 0.092 | | | 18.2646 | | | | | | 5.27712 | | | | ph_40 | |
| 86 | | | | | (D) | | | |  | | | - | | | | | | | | - | | | 0.192 | | | 18.08253 | | | | | | 4.59718 | | | | ph_42 | |
| 87 | | | | | (D) | | | |  | | | - | | | | | | | | - | | | 0.225 | | | 18.51318 | | | | | | 4.65706 | | | | ph_43 | |
| 88 | | | | | (D) | | | |  | | |  | | | | | | | | - | | | 0.048 | | | 18.96998 | | | | | | 4.58312 | | | | ph_44 | |
| 89 | | | | | (D) | | | |  | | |  | | | | | | | | - | | | 0.190 | | | 18.78865 | | | | | | 3.90318 | | | | ph_45 | |
| 90 | | | | | (D) | | | |  | | |  | | | | | | | |  | | | 0.230 | | | 19.21947 | | | | | | 3.96306 | | | | ph_46 | |
| 91 | | | | | (D) | | | |  | | |  | | | | | | | |  | | | 0.043 | | | 19.95034 | | | | | | 5.15412 | | | | ph_47 | |
| 92 | | | | | (D) | | | |  | | |  | | | | | | | |  | | | 0.116 | | | 19.7691 | | | | | | 4.47418 | | | | ph_48 | |
| **Quinolines_E** | | | | | | | | | | | | | | | | | | | | | **Quinolines_F** | | | | | | | | | | | | | | |  | |
|  | | | | | | | | | | | | | | | | | | | | |  | | | | | | | | | | | | | | | | |
| **#** | | | **scaffold** | | | | **R_1_** | | | | **R_2_** | | | | **R_3_** | | | | | | **IC_50_ (µM)** | | | | **Molar refractivity** | | | | | **LogP(o/w)** | | | | | **ID** | | |
| 93 | | | (E) | | | |  | | | | - | | | | - | | | | | | 0.266 | | | | 15.88452 | | | | | 5.02506 | | | | | ph_20 | | |
| 94 | | | (E) | | | |  | | | | - | | | | - | | | | | | 0.224 | | | | 15.88452 | | | | | 5.02506 | | | | | ph_22 | | |
| 95 | | | (E) | | | |  | | | | - | | | | - | | | | | | 0.248 | | | | 15.9959 | | | | | 3.678 | | | | | ph_24 | | |
| 96 | | | (E) | | | |  | | | | - | | | | - | | | | | | 0.165 | | | | 16.13804 | | | | | 4.405 | | | | | ph_25 | | |
| 97 | | | (E) | | | |  | | | | - | | | | - | | | | | | 0.405 | | | | 15.70624 | | | | | 3.98759 | | | | | ph_26 | | |
| 98 | | | (E) | | | |  | | | | - | | | | - | | | | | | 0.159 | | | | 16.55512 | | | | | 5.033 | | | | | ph_27 | | |
| 99 | | | (F) | | | |  | | | | - | | | | - | | | | | | 1.454 | | | | 15.9279 | | | | | 6.601 | | | | | ph_158 | | |
| 100 | | | (F) | | | |  | | | | - | | | | - | | | | | | 0.995 | | | | 15.9273 | | | | | 6.75753 | | | | | ph_159 | | |
| 101 | | | (F) | | | |  | | | | - | | | | - | | | | | | 0.613 | | | | 16.06989 | | | | | 5.606 | | | | | ph_160 | | |
| 102 | | | (F) | | | |  | | | | - | | | | - | | | | | | 1.077 | | | | 15.75251 | | | | | 5.95106 | | | | | ph_161 | | |
| **Quinolines_G** | | | | | | | | | | | **Quinolines_H** | | | | | | | | | | | | | | | **Quinolines_I** | | | | | | | | | | | |
|  | | | | | | | | | | |  | | | | | | | | | | | | | | |  | | | | | | | | | | | |
| **#** | | **scaffold** | | | | | | | **R_1_** | | | | | **R_2_** | | **R_3_** | |  | | | | | | **IC_50_ (µM)** | | | | | **Molar refractivity** | | | | **LogP**  **(o/w)** | | | | **ID** |
| 103 | | (G) | | | | | | |  | | | | | - | | - | |  | | | | | | 23.000 | | | | | 10.5935 | | | | 5.24853 | | | | ph_118 |
| 104 | | (G) | | | | | | |  | | | | | - | | - | |  | | | | | | 2.057 | | | | | 15.34394 | | | | 6.25753 | | | | ph_156 |
| 105 | | (G) | | | | | | |  | | | | | - | | - | |  | | | | | | 1.075 | | | | | 16.11326 | | | | 5.24453 | | | | ph_157 |
| 106 | | (H) | | | | | | |  | | | | | - | | - | |  | | | | | | 1.476 | | | | | 15.75251 | | | | 6.14659 | | | | ph_106 |
| 107 | | (I) | | | | | | |  | | | | | - | | - | |  | | | | | | 0.1330 | | | | | 18.44468 | | | | 6.00159 | | | | ph_73 |
| 108 | | (I) | | | | | | | - | | | | | - | | - | |  | | | | | | 0.9810 | | | | | 15.75311 | | | | 5.93359 | | | | ph_162 |
| **#** | | | | **scaffold** | | | |  | | **Quinoline** | | | | | | | | | | | | | | | | | **IC_50_ (µM)** | | | | **Molar refractivity** | | | **LogP**  **(o/w)** | | | **ID** |
| 109 | | | | (J) | | | |  | |  | | | | | | | | | | | | | | | | | 1.205 | | | | 15.51011 | | | 7.158 | | | ph_4 |
| 110 | | | | (K) | | | |  | |  | | | | | | | | | | | | | | | | | 0.827 | | | | 18.2646 | | | 5.277 | | | ph_41 |
| 111 | | | | (L) | | | |  | |  | | | | | | | | | | | | | | | | | 0.730 | | | | 19.30865 | | | 6.329 | | | ph_155 |
