# Supplementary material for: A 2D-QSAR and Grid-Independent Molecular Descriptor (GRIND) Analysis of Quinoline-Type Inhibitors of Akt2: Exploration of the Binding Mode in the Pleckstrin Homology (PH) Domain
Source: PLoS One. 2016 Dec 30;11(12):e0168806. doi: 10.1371/journal.pone.0168806 (PMC5201309; doi:10.1371/journal.pone.0168806)
Supplement: S2 Table — (DOCX) [file pone.0168806.s003.docx]

**S2 Table:** Experimental and predicted biological activity values (log(1/IC_50_)) of training and test sets obtained after Leave-One-Out (LOO) cross validation.

| **Training set** | | |  | **Test set** | | |  |
| --- | --- | --- | --- | --- | --- | --- | --- |
| **Comp**  **ID** | **Experimental (log(1/IC_50_))** | **Predicted**  **(log(1/IC_50_))** | **Residual Values=Exp-Pred** | **Comp**  **ID** | **Experimental (log(1/IC_50_))** | **Predicted**  **(log(1/IC_50_))** | **Residual Values=Exp-Pred** |
| ph_68 | 1.721 | 1.400 | 0.320 | ph_21 | 1.408 | 1.186 | 0.222 |
| ph_118 | -1.361 | -0.810 | -0.550 | ph_19 | -0.438 | 0.207 | -0.645 |
| ph_120 | -0.866 | -0.566 | -0.299 | ph_44 | 1.318 | 0.816 | 0.502 |
| ph_66 | 0.707 | 1.154 | -0.447 | ph_148 | 1.187 | 0.569 | 0.618 |
| ph_164 | 0.322 | -0.000 | 0.322 | ph_32 | 1.096 | 0.783 | 0.313 |
| ph_135 | 0.329 | 0.455 | -0.125 | ph_58 | 0.903 | 0.767 | 0.136 |
| ph_83 | -0.113 | -0.565 | 0.451 | ph_36 | 0.886 | 1.093 | -0.206 |
| ph_14 | 0.605 | 0.148 | 0.456 | ph_78 | 0.841 | 0.569 | 0.272 |
| ph_165 | 0.575 | 0.33 | 0.236 | ph_42 | 0.716 | 0.729 | -0.012 |
| ph_84 | -0.950 | -0.178 | -0.776 | ph_60 | 0.701 | 0.888 | -0.186 |
| ph_64 | 0.164 | 0.430 | -0.265 | ph_74 | 0.677 | 0.062 | 0.615 |
| ph_35 | 0.835 | 0.897 | -0.061 | ph_153 | 0.677 | 0.062 | 0.615 |
| ph_155 | 0.136 | 0.418 | -0.282 | ph_46 | 0.638 | 0.868 | -0.229 |
| ph_76 | 0.411 | 0.108 | 0.302 | ph_20 | 0.575 | 0.612 | -0.036 |
| ph_114 | 0.315 | 0.542 | -0.226 | ph_80 | 0.559 | 0.471 | 0.088 |
| ph_97 | -0.196 | -0.067 | -0.128 | ph_75 | 0.553 | 0.108 | 0.443 |
| ph_81 | -0.257 | -0.463 | 0.205 | ph_146 | 0.551 | 0.108 | 0.443 |
| ph_90 | -1.242 | -0.465 | -0.776 | ph_152 | 0.522 | -0.027 | 0.549 |
| ph_11 | -1.113 | -0.555 | -0.558 | ph_3 | 0.488 | -0.395 | 0.883 |
| ph_137 | -1.195 | -0.664 | -0.531 | ph_151 | 0.478 | 0.566 | -0.087 |
| ph_96 | -0.723 | -0.566 | -0.157 | ph_147 | 0.411 | 0.108 | 0.303 |
| ph_156 | -0.313 | -0.305 | -0.007 | ph_41 | 0.082 | 0.822 | -0.739 |
| ph_111 | -0.083 | 0.789 | -0.873 | ph_104 | 0.046 | 0.094 | -0.047 |
| ph_119 | 0.677 | 0.513 | 0.164 | ph_154 | 0.045 | 0.062 | -0.016 |
| ph_51 | 0.335 | 0.614 | -0.279 | ph_162 | 0.008 | 0.101 | -0.092 |
| ph_72 | 1.568 | 1.466 | 0.102 | ph_159 | 0.002 | 0.037 | -0.034 |
| ph_145 | 0.488 | -0.297 | 0.785 | ph_161 | -0.032 | 0.043 | -0.075 |
| ph_150 | -0.273 | 0.565 | -0.839 | ph_1 | -0.313 | -0.305 | -0.008 |
| ph_16 | 0.374 | -0.274 | 0.648 | ph_163 | -0.428 | 0.083 | -0.511 |
| ph_56 | 0.995 | 0.792 | 0.202 | ph_71 | 1.408 | 1.186 | 0.223 |
| ph_24 | 0.605 | 0.498 | 0.106 |  |  |  |  |
| ph_92 | 0.306 | 0.390 | -0.083 |  |  |  |  |
| ph_34 | 0.546 | 0.762 | -0.215 |  |  |  |  |
| ph_7 | -0.682 | -0.496 | -0.185 |  |  |  |  |
| ph_107 | 0.522 | 0.541 | -0.018 |  |  |  |  |
| ph_110 | 0.968 | 0.637 | 0.331 |  |  |  |  |
| ph_39 | 1.167 | 1.207 | -0.040 |  |  |  |  |
| ph_121 | -0.314 | -0.588 | 0.274 |  |  |  |  |
| ph_117 | 1.126 | 0.975 | 0.151 |  |  |  |  |
| ph_129 | 0.238 | 0.359 | -0.121 |  |  |  |  |
| ph_133 | -0.257 | -0.247 | -0.010 |  |  |  |  |
| ph_95 | -0.966 | -0.588 | -0.378 |  |  |  |  |
| ph_61 | 0.651 | 0.450 | 0.201 |  |  |  |  |
| ph_127 | -0.089 | -0.115 | 0.026 |  |  |  |  |
| ph_103 | -0.568 | 0.108 | -0.677 |  |  |  |  |
| ph_82 | 0.329 | 0.239 | 0.090 |  |  |  |  |
| ph_142 | -0.244 | -0.057 | -0.187 |  |  |  |  |
| ph_102 | 0.232 | 0.075 | 0.156 |  |  |  |  |
| ph_116 | 0.599 | 0.767 | -0.168 |  |  |  |  |
| ph_79 | 0.209 | 0.471 | -0.261 |  |  |  |  |
| ph_98 | -0.053 | -0.171 | 0.117 |  |  |  |  |
| ph_132 | 0.463 | 0.222 | 0.241 |  |  |  |  |
| ph_93 | 0.276 | -0.077 | 0.354 |  |  |  |  |
| ph_69 | 0.133 | 0.745 | -0.611 |  |  |  |  |
| ph_2 | 0.677 | 0.062 | 0.615 |  |  |  |  |
| ph_8 | -0.822 | -0.928 | 0.105 |  |  |  |  |
| ph_47 | 1.3665 | 1.126 | 0.240 |  |  |  |  |
| ph_43 | 0.647 | 0.875 | -0.227 |  |  |  |  |
| ph_115 | 0.424 | -0.098 | 0.523 |  |  |  |  |
| ph_109 | 0.775 | 0.581 | 0.193 |  |  |  |  |
| ph_4 | -0.081 | -0.522 | 0.441 |  |  |  |  |
| ph_31 | 0.732 | 0.903 | -0.171 |  |  |  |  |
| ph_52 | 0.640 | 0.479 | 0.160 |  |  |  |  |
| ph_62 | 0.375 | 0.824 | -0.448 |  |  |  |  |
| ph_125 | -0.866 | -0.326 | -0.540 |  |  |  |  |
| ph_27 | 0.798 | 0.867 | -0.069 |  |  |  |  |
| ph_50 | 0.411 | 0.077 | 0.334 |  |  |  |  |
| ph_65 | 0.838 | 1.023 | -0.184 |  |  |  |  |
| ph_73 | 0.876 | 0.814 | 0.061 |  |  |  |  |
| ph_100 | 0.229 | 0.166 | 0.063 |  |  |  |  |
| ph_128 | 0.076 | 0.257 | -0.181 |  |  |  |  |
| ph_88 | -0.064 | -0.351 | 0.286 |  |  |  |  |
| ph_45 | 0.721 | 0.722 | -0.001 |  |  |  |  |
| ph_38 | 1.008 | 1.073 | -0.064 |  |  |  |  |
| ph_131 | -0.706 | 0.044 | -0.750 |  |  |  |  |
| ph_149 | 0.841 | 0.569 | 0.272 |  |  |  |  |
| ph_37 | 0.853 | 1.239 | -0.385 |  |  |  |  |
| ph_91 | -0.253 | -0.149 | -0.103 |  |  |  |  |
| ph_130 | 0.562 | 0.190 | 0.371 |  |  |  |  |
| ph_67 | 0.752 | 0.996 | -0.244 |  |  |  |  |
| ph_28 | 1.397 | 0.789 | 0.608 |  |  |  |  |
| ph_134 | -0.078 | 0.444 | -0.523 |  |  |  |  |
| ph_160 | 0.212 | -0.068 | 0.281 |  |  |  |  |
| ph_101 | -0.360 | 0.229 | -0.590 |  |  |  |  |
| ph_106 | -0.169 | -0.098 | -0.070 |  |  |  |  |
| ph_63 | 1.154 | 0.653 | 0.501 |  |  |  |  |
| ph_22 | 0.649 | 0.612 | 0.037 |  |  |  |  |
| ph_113 | 0.753 | 0.582 | 0.171 |  |  |  |  |
| ph_6 | -0.496 | -0.635 | 0.138 |  |  |  |  |
| ph_87 | -0.140 | -0.287 | 0.147 |  |  |  |  |
| ph_13 | 0.804 | 0.030 | 0.774 |  |  |  |  |
| ph_9 | -1.057 | -0.928 | -0.129 |  |  |  |  |
| ph_25 | 0.782 | 0.664 | 0.117 |  |  |  |  |
| ph_18 | -0.291 | 0.110 | -0.401 |  |  |  |  |
| ph_89 | -0.180 | -0.286 | 0.106 |  |  |  |  |
| ph_157 | -0.031 | 0.017 | -0.048 |  |  |  |  |
| ph_53 | 0.614 | 0.513 | 0.101 |  |  |  |  |
| ph_15 | -0.235 | -0.262 | 0.026 |  |  |  |  |
| ph_126 | 0.254 | -0.201 | 0.456 |  |  |  |  |
| ph_33 | 0.744 | 0.928 | -0.183 |  |  |  |  |
| ph_54 | 0.774 | 0.507 | 0.267 |  |  |  |  |
| ph_30 | 0.725 | 0.769 | -0.043 |  |  |  |  |
| ph_86 | -0.232 | -0.300 | 0.067 |  |  |  |  |
| ph_48 | 0.935 | 1.033 | -0.097 |  |  |  |  |
| ph_40 | 1.036 | 0.822 | 0.213 |  |  |  |  |
| ph_105 | 0.575 | 0.773 | -0.198 |  |  |  |  |
| ph_144 | -0.654 | -0.325 | -0.329 |  |  |  |  |
| ph_49 | 0.046 | 0.094 | -0.048 |  |  |  |  |
| ph_122 | -0.910 | -0.452 | -0.457 |  |  |  |  |
| ph_26 | 0.392 | 0.656 | -0.263 |  |  |  |  |
| ph_70 | 1.387 | 0.875 | 0.511 |  |  |  |  |
| ph_57 | 1.356 | 0.767 | 0.589 |  |  |  |  |
| ph_77 | 1.187 | 0.569 | 0.617 |  |  |  |  |
| ph_99 | 0.197 | 0.290 | -0.092 |  |  |  |  |
| ph_85 | -0.094 | -0.367 | 0.273 |  |  |  |  |
| ph_158 | -0.162 | 0.004 | -0.167 |  |  |  |  |
| ph_55 | 0.735 | 0.631 | 0.103 |  |  |  |  |
| ph_10 | -0.941 | -0.976 | 0.035 |  |  |  |  |
| ph_17 | -0.176 | -0.311 | 0.134 |  |  |  |  |
| ph_136 | -0.320 | -0.305 | -0.014 |  |  |  |  |
| ph_59 | 1.284 | 0.882 | 0.401 |  |  |  |  |
| ph_141 | -0.071 | -0.501 | 0.429 |  |  |  |  |
| ph_29 | 1.075 | 0.935 | 0.140 |  |  |  |  |
